# Supplementary material for: Abuse of alcohol among farmers: Prevalence and associated factors
Source: PLoS One. 2021 Aug 5;16(8):e0254904. doi: 10.1371/journal.pone.0254904 (PMC8341596; doi:10.1371/journal.pone.0254904)
Supplement: Attachment — Submitted filename: Response to Reviewers.pdf [file pone.0254904.s001.pdf]

Dear Sirs,

We are pleased to resubmit for your consideration the revised version of the manuscript PONE-D-21-02257 entitled 'Abuse of alcohol among farmers: prevalence and associated factors'. We address each of their comments as described below.

1. Please include in your Methods section the date ranges during which you recruited participants for this study.

In the Methods section, your suggested changes have been accommodated and the date ranges during which you recruited participants for this study are included, adjusting the text for clarity (page 03, paragraph 01).

2. Include your tables as part of your main manuscript and remove the individual files. Note that supplemental tables (should remain / be uploaded) as separate "supporting information" files.

Tables were included in the text at the time they were cited.

Table 01 (page 07); Table 02 (page 08); Table 03 (page 09) and Table 04 (page 10).

3. We note that Figure (s) 1 in your submission contains map images that may be copyrighted. You may request permission from the original copyright holder of Figure (s) 1 to publish the content specifically under the CC BY 4.0 license.

This figure was prepared especially for the composition of the article submitted in the plos one journal, and has not been previously presented or published in any other medium, as per the request form for permission to publish content under CC-BY license, also attached.

Reviewer comments:

Reviewer #1: The author presents the results of a cross-sectional study where he aimed to identify the prevalence and risk factors for alcohol abuse among a group of farmers in northeastern Brazil. The article adds new statistics and identifies risk factors in the field of alcohol abuse among farmers. The experimental and statistical methods described in the article are sound and the conclusions are supported by the data presented.

The short length of each paragraph is commendable. The authors attempt to write the article in plain English. Despite their best efforts, however, I strongly recommend that the article be checked for grammar and sentence clarity, if possible, by a native English speaker.

We welcome your comments.

Our manuscript has been revised to improve readability.

Reviewer #2: This is a well written article. The CAGE methodology is indeed a very important and useful questionnaire. However, other than merely stating the known facts about the prevalence of alcohol abuse in society, this manuscript does not add any new information to the existing body of literature.

Although there are published studies on alcohol consumption and alcohol-related problems in rural regions of several countries, the body of literature on this topic, in farmers in Brazil, is still insufficient. Moreover, the prevalence of alcohol abuse found in this study was higher than that found in other rural locations in Brazil.

The results reinforce the need for research to design effective strategies to address vulnerable populations and prevent alcohol abuse, and to improve living and working conditions. Furthermore, it reinforces the need for strategies that direct the reformulation of alcohol public policies aimed at promoting the health of vulnerable groups, such as the rural population and, especially, farmers.

Reviewer # 3: The manuscript by ALVES et al describes the prevalence and factors associated with alcohol consumption among farmers in the municipality of Caicó / RN and indicates the need for social support for this group of workers in the context of occupational health. The manuscript is well written and easy to follow. However, presenting the results in a tabular form makes it easier for readers to follow. Present the data from the univariate and multivariate analysis in a table.

The results suggest that 70% of the study population has a family member diagnosed with a mental disorder. This was also significant in the multivariate analysis. This factor would certainly have contributed to the subjects of the study consuming alcohol. But, I am just wondering why their family members have a high percentage of mental problems and what factors would have contributed to this? One reason I could think of is their socioeconomic status, but this factor was not significantly associated with the high prevalence of alcohol abuse. was there a gender difference in family members with a diagnosis of mental disorder?

We agree with your analysis, we also believe that the fact that family members have mental disorders can contribute to alcohol consumption. Furthermore, we also agree that this characteristic may be associated with the socioeconomic condition, since poverty, unemployment, debt, or loss of socioeconomic capacity are associated with the emergence of psychological suffering and/or worsening of mental disorders, especially depression, anxiety, suicide, and consumption of alcohol and other drugs. We also add health and labor factors, since the difficulty of access to health services for rural residents (the majority of the population in our study), especially mental health services, access to psychology and psychiatry professionals.

We agree that this explanation is speculative at the moment, and we have edited the text to suggest also that this hypothesis can be considered by our results.
